# Influence of silicon on growth mechanism of micro-arc oxidation coating on cast Al–Si alloy

**DOI:** 10.1098/rsos.172428

**Published:** 2018-07-04

**Authors:** Huijun Yu, Qing Dong, Yang Chen, Chuanzhong Chen

**Affiliations:** 1 Key Laboratory of High-efficiency and Clean Mechanical Manufacture (Shandong University), Ministry of Education, School of Mechanical Engineering, Shandong University, Ji'nan 250061, Shandong, People's Republic of China; 2 National Demonstration Center for Experimental Mechanical Engineering Education (Shandong University), School of Mechanical Engineering, Shandong University, Ji'nan 250061, Shandong, People's Republic of China; 3 Key Laboratory for Liquid-Solid Structural Evolution and Processing of Materials (Ministry of Education), School of Materials Science and Engineering, Shandong University, Ji'nan 250061, Shandong, People's Republic of China

**Keywords:** Al–Si alloys, micro-arc oxidation, coating, growth process

## Abstract

Micro-arc oxidation (MAO) is a plasma-assisted electrochemistry method to prepare protective ceramic coatings on aluminium alloys. Alloy elements of the Al-alloy substrate, such as Si, Cu, Mg and Li, have effects on the microstructure and composition of the MAO coatings. Usually, silicon distributes in the cast Al–Si alloy substrate as small laths and they cover approximately 10% of the substrate surface. Therefore, their effects on the growth process and microstructure of the MAO coatings are worthy of notice. In the present study, oxide coatings with a thickness of 15–18 µm were prepared on the ZL109 Al–Si alloy by MAO. The phase content, surface morphology and element distribution of the coatings were investigated by X-ray diffraction, grazing incidence X-ray diffraction, scanning electron microscope, and electron probe micro-analysis respectively. The average hardness of the coatings was 622.3 ± 10.2 HV_0.05_. The adhesive strength of the coatings is 40.55 ± 2.55 N, and the adhesion of the coatings could be rated as 5B by tape test according to ASTM D3359-17 standard test methods, which indicated a high adhesive strength between the MAO coating and substrate. The effects of silicon laths on surface morphology and composition of the coatings were discussed, and a model was put forward to describe the growth process of the MAO coatings on cast Al–Si alloys. The authors believe that the high silicon content of the substrate has no adverse influence on the structure and properties of the MAO coating on the ZL109 alloy.

## 1. Introduction

Micro-arc oxidation (MAO), also named as plasma electrolysis oxidation, is a plasma-assisted electrochemistry method that can be used to prepare protective ceramic coatings on aluminium alloys [1–4]. A key feature of the process is a plasma discharge that occurs at the metal/electrolyte interface when the applied voltage exceeds a certain critical breakdown value (typically several hundreds of volts) and appears as a number of discrete short-lived microdischarges moving across the metal surface [5–7]. After the MAO treatment, hard and well-adhered oxide coatings with excellent tribological performance and dielectric properties can be prepared on the metal surface [8–10]. It is thought that the MAO technology is an effective and promising surface strengthening method for aluminium and its alloys [11].

Cast Al–Si alloys, usually with silicon content *ω*_Si_ > 10%, are the most commonly used aluminium alloys in the modern automobile industry [12,13]. Surface protection is required for many parts, such as the piston, piston ring and connecting rod in the engine, that often encounter early failure caused by the surface abrasion and corrosion [14]. Many attempts to prepare MAO coatings on Al–Si alloys have been reported [15–17]. As the substrate has a high content of silicon and the areas covered by silicon laths take approximately 10% of the substrate surface, its effects on the MAO process and coating composition are worthy of notice. In previous studies, coatings on these alloys were considered as a mixture of Al–Si–O compounds dominated by γ-Al_2_O_3_ or α-Al_2_O_3_ [16,18,19]. Small amounts of silicon compounds such as Al_2_SiO_5_ and mullite were found as well [16,20]. Wang *et al*. had made a model to describe the growth process of the MAO coatings [15]. However, they ignored a fact that the coatings grow towards the inside of the substrate even at the beginning of the MAO process. So in this paper, similar MAO coatings were made on the ZL109 alloy in a phosphate electrolyte. The effects of silicon laths in the substrate on the coating growth and composition were investigated and a new model was put forward to describe the growth process of the MAO coatings on cast Al–Si alloys.

## 2. Experimental procedures

The cast Al–Si alloy named ZL109, with a nominal composition of 11–13% Si, 0.5–1.5% Cu, 0.8–1.5% Mg and Al balance, was used as the substrate material. Rectangular samples (12 × 8 × 4 mm^3^) were ground using 1000# SiC paper, based on our experience, this will normally deliver an average roughness of around 1 micron. Then the samples were cleaned with acetone and distilled water before the MAO treatment. For the MAO treatment, the specimen that was used as an anode was immersed in a solution of phosphate salt (0.6 mol l^−1^ Na_3_PO_4_, pH = 8), while a stainless steel plate was used as a cathode. Electrolyte temperature was controlled under 30°C. The current density was in the range of 4–6 A dm^−2^. The duration for the MAO treatment was 30 min. After the MAO treatment, samples were washed with distilled water and dried at room temperature. In order to evaluate the effect of Si on the microdischarge in the MAO progress, a pure aluminium sample containing 99.99% Al was also involved.

The phase contents of the MAO coating were determined by X-ray diffraction (XRD, D/max-RC, *i *= 40 mA, 4° min^−1^) and grazing incidence X-ray diffraction (GIXD, X’ Pert PRO, *i *= 35 mA, 3° min^−1^), both using Cu-K*_α_* radiation operated at a voltage of 40 kV. The incidence angle for GIXD is 0.5°. The coating surface and cross-section micrographs were observed by JXA-8800R electron probe micro-analysis (EPMA). Element distributions in the MAO coating were also analysed. The hardness and adhesive strength of the coatings were evaluated using Shimadzu Vickers hardness tester, WP-2000 scratch tester and ASTM D3359-17 standard test methods for rating adhesion by tape test. 3M 610-1PK-25.4 mm tape (Scotch^®^) was used in the tape test. For the scratch test, the loading rate was 40 N min^−1^, the probe speed was 4 mm min^−1^ and the maximum load was 80 N sliding. The micrographs of the scratch test were observed by JSM-6380 scanning electron microscope (SEM). Owing to the low conductivity of the coating, the samples were sputter-coated with carbon prior to the EPMA and SEM observations.

## 3. Results

### 3.1 Phase analysis

**Figure 1.**
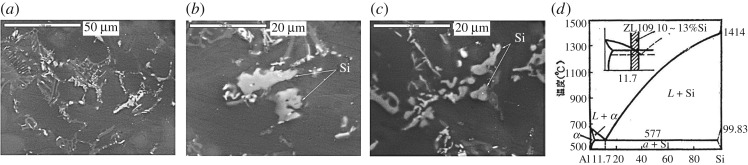
Si phase distributions of ZL109 alloy, as shown in (*a*) and (*b*,*c*) at low and high magnification, respectively; (*d*) Al–Si phase diagram.

The ZL109 alloy is a eutectic Al–Si alloy (alloys with lesser than 11.7% Si, the eutectic composition) whose microstructure contains coarse α-Al dendrites and flake-like eutectic silicon phases according to the Al–Si phase diagram [21,22], as shown in figure 1. Si improves the fluidity and castability of aluminium [23]. The combined area covered with silicon takes more than 10% of the substrate surface, and the size of silicon particles is in the range of several to several tens of microns.

**Figure 2.**
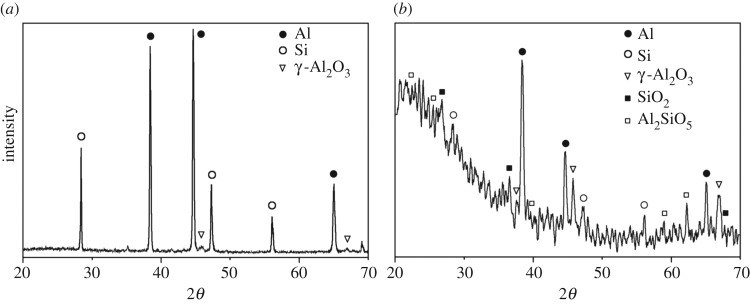
Phase analysis of MAO coatings on ZL109 alloy in a solution of phosphate salt by: (*a*) XRD and (*b*) GIXD.

The XRD pattern of the MAO coating on the Al–Si alloy is shown in figure 2*a*. Aluminium and silicon with a small amount of γ-Al_2_O_3_ can be identified. As the Al–Si alloy substrate used in this work is a typical eutectic structure of aluminium solid solution that contains small silicon particles, the authors believe that X-ray could probably penetrate through the thin MAO coating and the peaks of aluminium and silicon are most likely to come from the substrate. Therefore, GIXD is used to give a clear result, as shown in figure 2*b*. It can be seen that the peaks of γ-Al_2_O_3_ become sharper and clearer, while the peak strength of aluminium and silicon obviously decreases. This result confirms the authors' inference and indicates that the coating is mainly composed of γ-Al_2_O_3_. In addition, small amounts of other phases, SiO_2_ (quartz) and Al_2_SiO_5_ are identified from figure 2*b*.

### 3.2 Surface, cross-section morphology and element distributions

**Figure 3.**
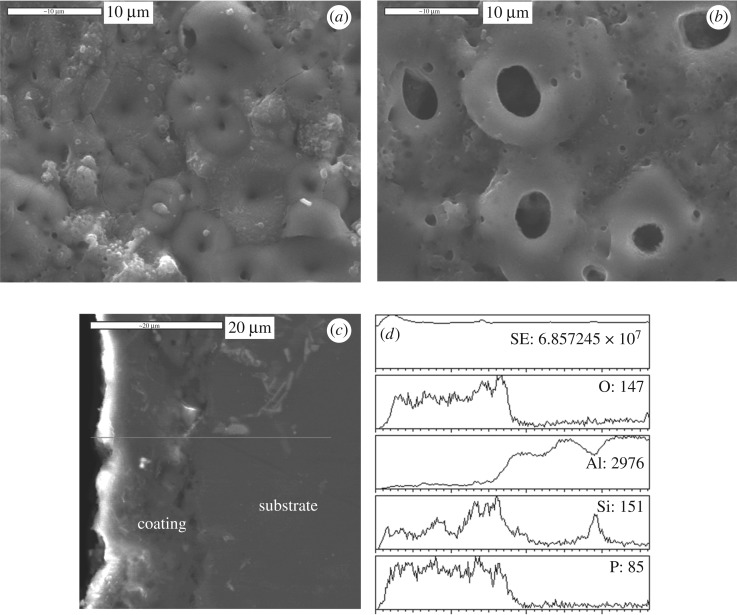
Surface and cross-section micrographs of MAO coatings: (*a*) on pure aluminium, (*b*) on ZL109 alloy; (*c*) is cross-section micrograph for the coating on ZL109 alloy and (*d*) is the pattern of element line analysis.

Figure 3 shows the surface and cross-section micrographs of the MAO coating on pure aluminium (figure 3*a*) and the Al–Si alloy (figure 3*b*). Both of their surfaces, over which round pores distribute evenly, are a little rugged. But the surfaces are quite different in pore size and density. The pores on the Al–Si alloy are larger, with diameters of 3.61 ± 1.31 µm and a low density; while the pores on pure aluminium are smaller, with diameters of 1.06 ± 0.47 µm and a relatively high density. The diameters of pores are measured from surface micrographs of MAO coatings. Usually, it is believed that the formation of the pores is attributed to the electrical discharge phenomenon occurring on the sample surface when voltage applied during the MAO process reaches a critical value (up to several hundred volts). In this condition, local areas of the sample surface can be broken down by an instantaneous micro-arc/spark. When the micro-arc/spark extinguishes, a pore would be left. But the porosity and hardness of MAO coatings could be optimized by adjusting MAO parameters (current density, composition of electrolyte and oxidation time) [24], and further sealing treatment would also make up the porous defects of the MAO layer [25]. Zhu *et al*. [26] sealed the pores of MAO coatings with grease, and it was proved that the sealed MAO coating exhibited a better fretting wear resistance and longer service life. Ivanou *et al*. [27] sealed the pores of MAO coatings with hybrid epoxy-silane formulation, the sealed MAO coating provided good corrosion resistance.

The cross-section micrograph and element distributions in direction of depth of the MAO coating on the ZL109 alloy are shown in figure 3*c*. It can be seen that the whole coating, with a thickness of 8–10 µm, is relatively dense and homogeneous in the direction of depth, which is quite different from the coating surface. There is no obvious crack in the coating. No discontinuous zone is observed between the coating and substrate. This implies that the MAO coating has high adhesive strength to the substrate. Figure 3*d* shows that the coatings on the Al–Si alloy are mainly composed of Al, O and Si with a trace amount of P, which is consistent with the XRD analysis.

### 3.3 Hardness and adhesive strength

The average hardness of the coating is 622.3 ± 10.2 HV_0.05_, which is about 7.9 times higher than that of the substrate (79.2 ± 1.2 HV_0.05_). The coating is mainly composed of γ-Al_2_O_3_ and small amounts of SiO_2_ and Al_2_SiO_5_. Student *et al*. [28] demonstrated that a proportion of 60% γ-Al_2_O_3_ in coatings exhibits high hardness. Krishna *et al*. [29] reported that hardness value of γ-Al_2_O_3_ phases is calculated to be 806 HV. The intrinsic hardness of the SiO_2_ is slightly lower than that of Al_2_O_3_ [30], but the hardness values of Al_2_SiO_5_ is higher than Al_2_O_3_ [31]. Therefore, within the experimental scope, the high hardness of our MAO coatings could be attributed to the formation of γ-Al_2_O_3_. But the eventual hardness of the coatings depends on the proportion of these phases. The high hardness of the coatings proved that micro-arc oxidation has the potential to prepare wear-resistance coatings on Al–Si alloys.

**Figure 4.**
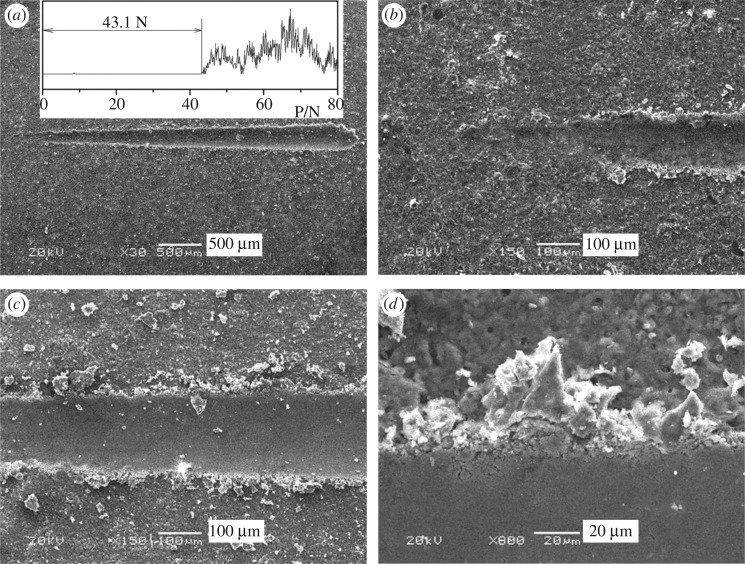
Friction-load curve and scratch micrographs of MAO coating on ZL109 alloy: (*a*) is the friction-load curve and whole track; (*b*), (*c*) and (*d*) are the beginning, midst and edge of the track, respectively.

**Figure 5.**
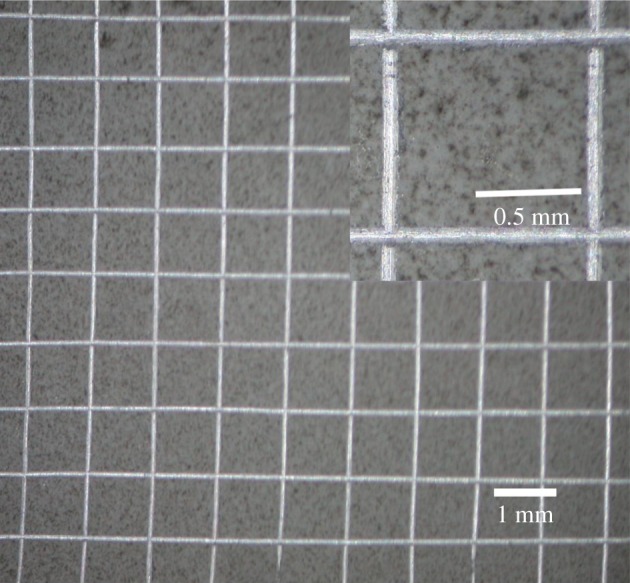
Surface morphology of cross-cut area of MAO coating on ZL109 alloy after tape test.

**Figure 6.**
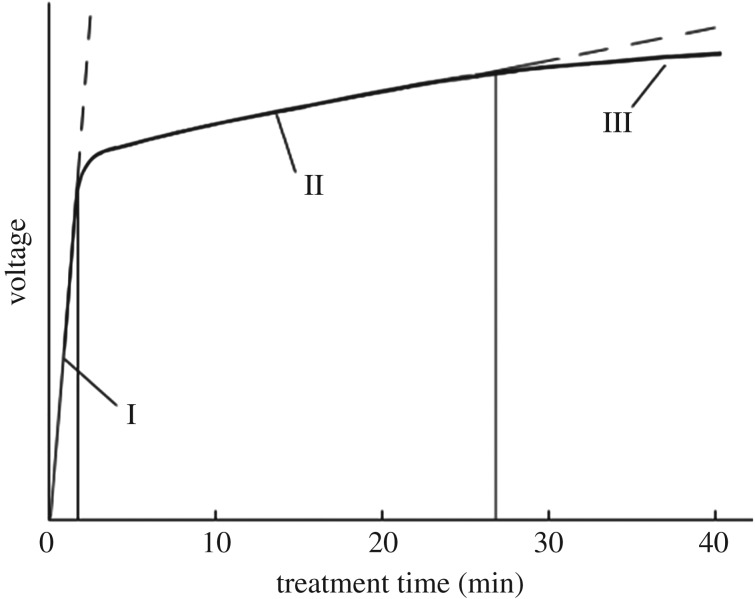
Schematic illustration of applied voltage at different times during the MAO process.

The friction-load curve of the MAO coating on the Al–Si alloy is shown in figure 4*a*. The adhesive strength of the coatings is 40.55 ± 2.55 N. The peak value is 43.1 N, which indicates that the MAO coating has high adhesive strength to the substrate. Figure 6 shows the micrographs of the scratch track of the MAO coating on the Al–Si alloy. The coating is smoothed and the coating material is extruded to both sides along the track (figure 4*a*). At the beginning of the track, as shown in figure 4*b*, the surface pores of the MAO coating can still be seen. However, this surface character has gradually disappeared in the midst of the track, and the substrate is exposed finally (figure 4*c*). The edge of the track is shown in figure 4*d*. The coating material is piled at the track edge, and there is no large peeling off, which implies the coating has excellent toughness. Furthermore, according to the ASTM D3359-17 standard test methods for rating adhesion by tape test [32,33], three samples were tested and three faces of each sample were tested to avoid occasionality. The adhesion of the samples could all be classified as 5B, owing to the edges of the cuts which are completely smooth, and none of the squares of the lattice is detached (figure 5). 5B is the highest rank of adhesion strength.

These two methods both demonstrate excellent adhesive strength between the MAO coatings and ZL109 substrate which would be suitable for commercial applications. The *in situ* formation of MAO coatings promises the good adhesive strength to the substrate [34].

## 4. Discussion

The effects of alloy elements on the MAO process of aluminium alloys were continuously studied by researchers. Nykyforchyn *et al*. [2] found that element Li is helpful to enhance the hardness of the MAO coating and the total fraction of Cu and Mg must be at least 3.5%. However, independent phases containing these elements were seldom observed in MAO coatings. References [8,35,36] showed that small amounts of phases containing silicon, such as Al_2_SiO_5_, Al_4_Si and Al_6_Si_2_O_13_ could be identified from the XRD patterns of MAO coatings on aluminium alloys, which implied the effect of silicon on the composition of the coatings. Compared with aluminium alloys with low alloy content, the oxidation process on cast Al–Si alloy is special, given that the mass fraction of silicon in the substrate is more than 10 wt.%. Many small silicon particles are distributed on the substrate of the Al–Si alloy. In other words, the combined area covered with silicon takes about 10% of the substrate surface. So what role would the small particles on the sample surface play in the MAO process? Can oxide coatings be formed on these silicon-covered areas? If it could, what structure and composition would this kind of oxide coating have? As the microdischarge is generated in a very small local area (with a diameter of 1–10 µm [6,37]), and its lifetime is very short, probably *t* < 1 ms [38], it is difficult to solve these problems by direct experimental observation. Therefore, a growth model of the MAO coating on Al–Si alloys was built by Wang *et al*. [15]. In their model, the voltage variation with treatment time and corresponding surface morphology and composition of the MAO-coated Al–Si alloys were considered. They believed that after the first stage of oxidation, namely the passivation stage, large micro-arc discharges first formed at the boundary of Al–Si. This happens when the voltage reaches a critical value owing to the tip/corner effect of electrical concentration on the localized surface where the interface of Al matrix and silicon grain exists. While passivation films grow towards the substrate in the passivation stage [39], there are some volume dilatations when Al and Si are transformed into their corresponding oxides (47% and 88%, respectively). So supposing that a surface layer of 10 nm thickness of the substrate was oxidized in the passivation stage, the tip height at the interface of different oxide areas would be only 4 nm, which is far smaller than substrate roughness (on an order of magnitude of microns [8,15,40]). So we believe that discharges cannot be generated by the tip/corner effect. Considering the surface morphology, phase content and composition of the MAO coatings, and comparing them with that of a pure aluminium, a new model to describe the growth of the MAO coating on Al–Si alloys is put forward. The MAO process was divided into three stages for succinctness, as shown in figure 6.

### 4.1 The passivation stage (I)

**Figure 7.**
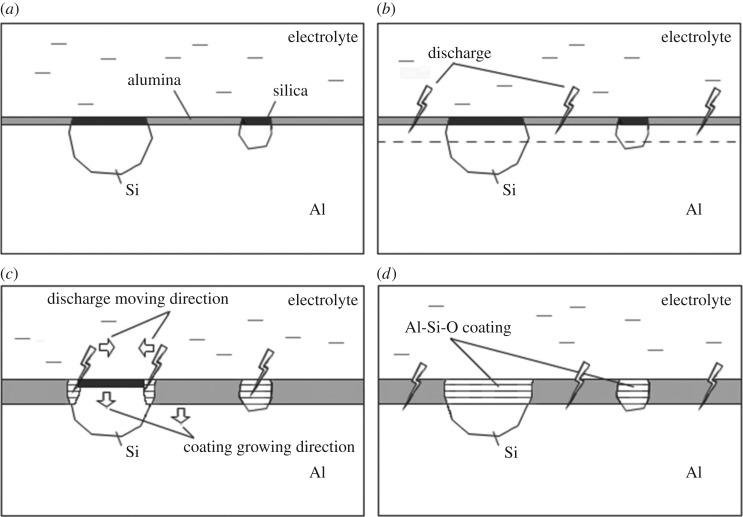
Schematic illustration of MAO coating growth on cast Al–Si alloy surface.

This is the stage before the applied voltage reaches the critical discharge potential. From reference [39], a passivation layer with a thickness of 6–20 nm can be formed on the exposed silicon crystals via chemisorption. A natural oxide layer on aluminium matrix, which is composed of amorphous Al_2_O_3_ with a thickness of 2–3 nm, also exists before passivation [6,41,42]. In the passivation stage, both of them will grow thicker and reach 10–15 nm by anodizing oxidation, as shown in figure 7*a*. The authors believe that there is no obvious difference in the thickness between the passivation oxide layers on aluminium matrix and silicon laths because the duration of this stage is very short (*t*_1_ < 2 min [15]).

### 4.2 The stable oxidation stage (II)

This is the typical MAO stage. When the voltage reaches the critical discharge potential, the thin oxide layers formed in the passivation stage will be broken down, and many tiny sparks around the sample can be seen [43–45]. Electrical properties of the main substances of the Al–Si alloy surface are shown in table 1 (compiled from references [8,34]). It can be seen that the dielectric strength of silica is 1.5–2.5 times higher than that of alumina. Therefore, alumina layers on aluminium matrix will first be broken down as the voltage increases (figure 7*b*). In the local discharge area, the passivation films are melted at the instantaneous high temperature and the aluminium matrix will further be oxidized by element diffusion. When a spark extinguishes, the local film will be rebuilt with the melted oxide that is quickly cooled by the electrolyte. As a result, the oxide film grows into the substrate and the discharge potential increases with thickness of the film. When the discharge voltage of alumina film becomes higher than that of the silica film, the discharge locations will change.

**Table 1.** Summary of electrical properties of main substances on the Al–Si alloy surface [44,46].

substances	dielectric constant	electrical resistivity (Ω cm^−1^)	dielectric strength (kV mm^−1^)
aluminium, Al	—	2.9 × 10^−10^	—
alumina, Al_2_O_3_	9.9	10^14^	10
silicon, Si	—	∼10^−6^	—
silica, SiO_2_	3.9	10^10^–10^14^	15–25

From table 1, the conductivity of aluminium is much better than that of silicon. So the edges of larger silicon particles, namely the boundary between the alumina and silica layers, are closer to the aluminium matrix and become positions that electric current is easier to pass through. The discharge will first move to these areas where new Al–Si–O films, a mixture of alumina and silica, will be formed via oxidation and diffusion. Then the discharge positions will move towards the centre of the silicon particles as the Al–Si–O films grow. For smaller silicon particles, discharges may happen on its entire areas, and the Al–Si–O films will be directly formed, as shown in figure 7*c*. Finally, a homogeneous thicker coating can be formed on the Al–Si alloys, and the discharge positions will return to the surface of aluminium matrix because the discharge voltage of silica film becomes higher than that of the alumina film again, as shown in figure 7*d*.

The above-mentioned process may be repeated many times during the stage. The discharge voltage will gradually increase as the MAO coating grows thicker, as shown in figure 6. The fact that the pore size of the MAO coatings on the ZL109 alloy is larger than that on pure aluminium (figure 3) can be attributed to the existence of silicon. Because the discharges happen alternately in different local areas such as the aluminium matrix and silicon particles, the discharge area of the Al–Si alloys is at times smaller than that of pure aluminium, especially when silicon particles are the main discharge areas. So if the current density for both aluminium and Al–Si alloys is at the same level, the strength of an individual microdischarge for Al–Si alloys will be much higher than that of pure aluminium.

### 4.3 The final oxidation stage (III)

In this stage, the increase of the applied voltage starts to become slower. The number of sparks obviously decreases and the discharges gradually concentrate to the edge of the samples. Different substances in the MAO coating react further as the coating thickness increases. Usually, the thickness of silicon particles is several microns, a little thinner than that of the oxide film. So in areas where silicon particles are smaller, the aluminium matrix under the silicon particles will be oxidized when they are completely oxidized. Meanwhile, in areas of the aluminium matrix, the growing alumina films may reach the shallow-distributed silicon particles under the substrate surface. Both of the two functions promote reactions between silica and alumina. Moreover, owing to the diffusion effect caused by the repeated discharges, the composition transition between alumina and Al–Si–O layers gradually become more homogeneous.

In addition, owing to the repeated effects of melting and condensation in stages (II) and (III), the coating transforms from amorphous alumina to crystalline alumina. In the XRD analysis, the diffraction peaks of Al_2_O_3_ are relatively sharp, which indicates its high crystallinity.

## 5. Conclusion

(i) Oxide coatings with a thickness of 8–10 µm were deposited on the substrate of the ZL109 Al–Si alloy by MAO. The average hardness of the coating was 622.3 ± 10.2 HV_0.05_, which was 7.9 times higher than that of the substrate. The high hardness was attributed to the formation of γ-Al_2_O_3_ in the coating. The surface of the coating was porous, and the pore diameter was 3.61 ± 1.31 µm. The coating matrix was homogeneous and with few defects. There was a high adhesive strength between the MAO coating and substrate. The adhesive strength of the coatings is 40.55 ± 2.55 N, and the adhesion of the coatings is classified as 5B.
(ii) Silicon particles in the Al–Si alloy substrate have great effects on the growth process, surface morphology and composition of the MAO coating. The MAO process was divided into three stages, namely the passivation stage, stable oxidation stage and final oxidation stage. After the passivation stage, local alumina layers on the aluminium matrix surface would first be broken down owing to their lower dielectric strength. With the discharge voltage gradually increasing, the local aluminium and silicon areas were alternately broken down, and the MAO coating grew towards the substrate and became thicker. The coating was composed of Al–Si–O compounds, with Al_2_O_3_ the main phase and small amounts of other phases, such as SiO_2_ or Al_2_SiO_5_. The formation of Al–Si–O compounds can be attributed to the element diffusion and reactions in the MAO coating when local coating areas were broken down. The authors believed that the existence of silicon particles in the substrate had no adverse influence on the growth and properties of the MAO coating on the ZL109 alloy.

## Data accessibility

This article does not contain any additional data.

## References

[1] Kaseem M, Min JH, Ko YG 2017 Corrosion behavior of Al-1wt% Mg-0.85wt%Si alloy coated by micro-arc-oxidation using TiO2 and Na2MoO4 additives: role of current density. J. Alloy. Compd 723, 448–455. (10.1016/j.jallcom.2017.06.275)

[2] Shchedrina I, Rakoch AG, Henrion G, Martin J 2014 Non-destructive methods to control the properties of MAO coatings on the surface of 2024 aluminium alloy. Surf. Coat. Technol. 238, 27–44. (10.1016/j.surfcoat.2013.10.032)

[3] Xue W, Wang C, Li Y, Deng Z, Chen R, Zhang T 2002 Effect of microarc discharge surface treatment on the tensile properties of Al–Cu–Mg alloy. Mater. Lett. 56, 737–743. (10.1016/S0167-577X(02)00605-5)

[4] Tseng C, Lee J, Kuo T, Kuo S, Tseng K 2012 The influence of sodium tungstate concentration and anodizing conditions on microarc oxidation (MAO) coatings for aluminum alloy. Surf. Coat. Technol. 206, 3437–3443. (10.1016/j.surfcoat.2012.02.002)

[5] Erfanifar E, Aliofkhazraei M, Fakhr Nabavi H, Sharifi H, Rouhaghdam AS 2017 Growth kinetics and morphology of plasma electrolytic oxidation coating on aluminum. Mater. Chem. Phys. 185:162–175. (10.1016/j.matchemphys.2016.10.019)

[6] Zhang Y et al. 2017 Micro-structures and growth mechanisms of plasma electrolytic oxidation coatings on aluminium at different current densities. Surf. Coat. Technol. 321, 236–246. (10.1016/j.surfcoat.2017.04.064)

[7] Yerokhin AL, Snizhko LO, Gurevina NL, Leyland A, Pilkington A, Matthews A 2003 Discharge characterization in plasma electrolytic oxidation of aluminium. J. Phys. D Appl. Phys. 36, 2110–2120. (10.1088/0022-3727/36/17/314)

[8] Tian J, Luo Z, Qi S, Sun X 2002 Structure and antiwear behavior of micro-arc oxidized coatings on aluminum alloy. Surf. Coat. Technol. 154, 1–7. (10.1016/S0257-8972(01)01671-1)

[9] Nie X, Meletis EI, Jiang JC, Leyland A, Yerokhin AL, Matthews A 2002 Abrasive wear/corrosion properties and TEM analysis of Al2O3 coatings fabricated using plasma electrolysis. Surf. Coat. Technol. 149, 245–251. (10.1016/S0257-8972(01)01453-0)

[10] Wang P, Wu T, Xiao YT, Zhang L, Pu J, Cao WJ, Zhong XM 2017 Characterization of micro-arc oxidation coatings on aluminum drillpipes at different current density. Vacuum 142, 21–28. (10.1016/j.vacuum.2017.04.038)

[11] Li J, Zhang Q, Cai H, Wang A, Zhang J, Hua X 2013 Controlled deposition, electrical and electrochemical properties of electroless nickel layers on microarc oxidized magnesium substrates. Mater. Lett. 93, 263–265. (10.1016/j.matlet.2012.11.112)

[12] Ye H 2003 An overview of the development of Al-Si-alloy based material for engine applications. J. Mater. Eng. Perform. 12, 288–297. (10.1361/105994903770343132)

[13] Kang N, Coddet P, Liao H, Baur T, Coddet C 2016 Wear behavior and microstructure of hypereutectic Al-Si alloys prepared by selective laser melting. Appl. Surf. Sci. 378, 142–149. (10.1016/j.apsusc.2016.03.221)

[14] Sharma RA, Dwivedi DK 2005 Influence of silicon (wt.%) and heat treatment on abrasive wear behaviour of cast Al–Si–Mg alloys. Mater. Sci. Eng. A 408, 274–280. (10.1016/j.msea.2005.08.013)

[15] Wang L, Nie X 2006 Silicon effects on formation of EPO oxide coatings on aluminum alloys. Thin Solid Films 494, 211–218. (10.1016/j.tsf.2005.07.184)

[16] Gulec AE, Gencer Y, Tarakci M 2015 The characterization of oxide based ceramic coating synthesized on Al–Si binary alloys by microarc oxidation. Surf. Coat. Technol. 269, 100–107. (10.1016/j.surfcoat.2014.12.031)

[17] Samuel AM, Garza-Elizondo GH, Doty HW, Samuel FH 2015 Role of modification and melt thermal treatment processes on the microstructure and tensile properties of Al–Si alloys. Mater. Des. 80, 99–108. (10.1016/j.matdes.2015.05.013)

[18] Tran Q, Sun J, Kuo Y, Tseng C, He J, Chin T 2017 Anomalous layer-thickening during micro-arc oxidation of 6061 Al alloy. J. Alloy. Compd 697, 326–332. (10.1016/j.jallcom.2016.11.372)

[19] He J, Cai QZ, Luo HH, Yu L, Wei BK 2009 Influence of silicon on growth process of plasma electrolytic oxidation coating on Al–Si alloy. J. Alloy. Compd 471, 395–399. (10.1016/j.jallcom.2008.03.114)

[20] Xue W, Shi X, Hua M, Li Y 2007 Preparation of anti-corrosion films by microarc oxidation on an Al–Si alloy. Appl. Surf. Sci. 253, 6118–6124. (10.1016/j.apsusc.2007.01.018)

[21] Wang K, Jiang HY, Wang QD, Ye B, Ding WJ 2016 Nanoparticle-induced nucleation of eutectic silicon in hypoeutectic Al-Si alloy. Mater. Charact. 117, 41–46. (10.1016/j.matchar.2016.04.016)

[22] Zhang Y, Zheng H, Liu Y, Shi L, Xu R, Tian X 2014 Cluster-assisted nucleation of silicon phase in hypoeutectic Al–Si alloy with further inoculation. Acta Mater. 70, 162–173. (10.1016/j.actamat.2014.01.061)

[23] Ludwig TH, Schonhovd Dæhlen E, Schaffer PL, Arnberg L 2014 The effect of Ca and P interaction on the Al–Si eutectic in a hypoeutectic Al–Si alloy. J. Alloy. Compd 586, 180–190. (10.1016/j.jallcom.2013.09.127)

[24] Jayaraj RK, Malarvizhi S, Balasubramanian V 2017 Optimizing the micro-arc oxidation (MAO) parameters to attain coatings with minimum porosity and maximum hardness on the friction stir welded AA6061 aluminium alloy welds. Def. Technol. 13, 111–117. (10.1016/j.dt.2017.03.003)

[25] Yang W, Xu D, Wang J, Yao X, Chen J 2018 Microstructure and corrosion resistance of micro arc oxidation plus electrostatic powder spraying composite coating on magnesium alloy. Corros. Sci. 136, 174–179. (10.1016/j.corsci.2018.03.004)

[26] Zhu MH, Cai ZB, Lin XZ, Zheng JF, Luo J, Zhou ZR 2009 Fretting wear behaviors of micro-arc oxidation coating sealed by grease. Wear 267, 299–307. (10.1016/j.wear.2008.12.080)

[27] Ivanou DK, Starykevich M, Lisenkov AD, Zheludkevich ML, Xue HB, Lamaka SV, Ferreira MGS 2013 Plasma anodized ZE41 magnesium alloy sealed with hybrid epoxy-silane coating. Corros. Sci. 73, 300–308. (10.1016/j.corsci.2013.04.019)

[28] Student ММ, Dovhunyk VМ, Klapkiv МD, Posuvailo VМ, Shmyrko VV, Kytsya АР 2012 Tribological properties of combined metal-oxide–ceramic layers on light alloys. Mater. Sci. 48, 180–190. (10.1007/s11003-012-9489-7)

[29] Krishna LR, Gupta PSVN, Sundararajan G 2015 The influence of phase gradient within the micro arc oxidation (MAO) coatings on mechanical and tribological behaviors. Surf. Coat. Technol. 269, 54–63. (10.1016/j.surfcoat.2015.02.047)

[30] Oktar FN, Agathopoulos S, Ozyegin LS, Gunduz O, Demirkol N, Bozkurt Y, Salman S 2007 Mechanical properties of bovine hydroxyapatite (BHA) composites doped with SiO_2_, MgO, Al2O3, and ZrO_2_. J. Mater. Sci. Mater. Med. 18, 2137–2143. (10.1007/s10856-007-3200-9) 17619958

[31] Gaida NA et al. 2017 Synthesis of Al2O3/SiO2 nano-nano composite ceramics under high pressure and its inverse Hall-Petch behavior. J. Am. Ceram. Soc. 100, 323–332. (10.1111/jace.14551)

[32] ASTM. 2017 Standard test methods for rating adhesion by tape test. Designation: D3359–17. American Society for Testing and Materials.

[33] Monetta T, Acquesta A, Carangelo A, Donato N, Bellucci F 2017 Durability of AZ31 magnesium biodegradable alloys polydopamine aided: part 1. J. Magnes. Alloys 5, 412–422. (10.1016/j.jma.2017.09.006)

[34] Li Z, Di S 2017 Preparation and properties of micro-arc oxidation self-lubricating composite coatings containing paraffin. J. Alloy. Compd 719, 1–14. (10.1016/j.jallcom.2017.05.138)

[35] Matykina E, Arrabal R, Mohamed A, Skeldon P, Thompson GE 2009 Plasma electrolytic oxidation of pre-anodized aluminium. Corros. Sci. 51, 2897–2905. (10.1016/j.corsci.2009.08.004)

[36] Nie X, Leyland A, Song HW, Yerokhin AL, Dowey SJ, Matthews A 1999 Thickness effects on the mechanical properties of micro-arc discharge oxide coatings on aluminium alloys. Surf. Coat. Technol. 116–119, 1055–1060. (10.1016/S0257-8972(99)00089-4)

[37] Curran JA, Clyne TW 2006 Porosity in plasma electrolytic oxide coatings. Acta Mater. 54, 1985–1993. (10.1016/j.actamat.2005.12.029)

[38] Klapkiv MD, Klapkiv MD 1996 State of an electrolytic plasma in the process of synthesis of oxides based on aluminum. Mater. Sci. 31, 494–499. (10.1007/BF00559144)

[39] Wang H, Wang HW 2006 Analysis on porous aluminum anodic oxide film formed in Re–OA–H3PO4 solution. Mater. Chem. Phys. 97, 213–218. (10.1016/j.matchemphys.2005.03.061)

[40] Voevodin AA, Yerokhin AL, Lyubimov VV, Donley MS, Zabinski JS 1996 Characterization of wear protective Al Si O coatings formed on Al-based alloys by micro-arc discharge treatment. Surf. Coat. Technol. 86–87, 516–521. (10.1016/S0257-8972(96)03069-1)

[41] Doherty PE, Davis RS 1963 Direct observation of the oxidation of aluminum single-crystal surfaces. J. Appl. Phys. 34, 619–628. (10.1063/1.1729318)

[42] Thomas K, Roberts MW 1961 Direct observation in the electron microscope of oxide layers on aluminum. J. Appl. Phys. 32, 70–75. (10.1063/1.1735963)

[43] Pan YK, Chen CZ, Wang DG, Yu X, Lin ZQ 2012 Influence of additives on microstructure and property of microarc oxidized Mg–Si–O coatings. Ceram. Int. 38, 5527–5533. (10.1016/j.ceramint.2012.03.068)

[44] Kumar AM, Kwon SH, Jung HC, Shin KS 2015 Corrosion protection performance of single and dual plasma electrolytic oxidation (PEO) coating for aerospace applications. Mater. Chem. Phys. 149–150, 480–486. (10.1016/j.matchemphys.2014.10.049)

[45] Ono S, Moronuki S, Mori Y, Koshi A, Liao J, Asoh H 2017 Effect of electrolyte concentration on the structure and corrosion resistance of anodic films formed on magnesium through plasma electrolytic oxidation. Electrochim. Acta 240, 415–423. (10.1016/j.electacta.2017.04.110)

[46] Al Bosta MM, Ma K 2014 Influence of electrolyte temperature on properties and infrared emissivity of MAO ceramic coating on 6061 aluminum alloy. Infrared Phys. Technol. 67, 63–72. (10.1016/j.infrared.2014.07.009)

